# Gut Dysbiosis in Pancreatic Diseases: A Causative Factor and a Novel Therapeutic Target

**DOI:** 10.3389/fnut.2022.814269

**Published:** 2022-02-15

**Authors:** Tao Zhang, Guangqi Gao, Hafiz Arbab Sakandar, Lai-Yu Kwok, Zhihong Sun

**Affiliations:** ^1^Key Laboratory of Dairy Biotechnology and Engineering, Ministry of Education, Inner Mongolia Agricultural University, Hohhot, China; ^2^Key Laboratory of Dairy Products Processing, Ministry of Agriculture and Rural Affairs, Inner Mongolia Agricultural University, Hohhot, China; ^3^Inner Mongolia Key Laboratory of Dairy Biotechnology and Engineering, Inner Mongolia Agricultural University, Hohhot, China

**Keywords:** gut microbiome, pancreatic diseases, probiotics, prebiotics, synbiotics, postbiotics, fecal microbiota transplantation (FMT)

## Abstract

Pancreatic-related disorders such as pancreatitis, pancreatic cancer, and type 1 diabetes mellitus (T1DM) impose a substantial challenge to human health and wellbeing. Even though our understanding of the initiation and progression of pancreatic diseases has broadened over time, no effective therapeutics is yet available for these disorders. Mounting evidence suggests that gut dysbiosis is closely related to human health and disease, and pancreatic diseases are no exception. Now much effort is under way to explore the correlation and eventually potential causation between the gut microbiome and the course of pancreatic diseases, as well as to develop novel preventive and/or therapeutic strategies of targeted microbiome modulation by probiotics, prebiotics, synbiotics, postbiotics, and fecal microbiota transplantation (FMT) for these multifactorial disorders. Attempts to dissect the intestinal microbial landscape and its metabolic profile might enable deep insight into a holistic picture of these complex conditions. This article aims to review the subtle yet intimate nexus loop between the gut microbiome and pancreatic diseases, with a particular focus on current evidence supporting the feasibility of preventing and controlling pancreatic diseases *via* microbiome-based therapeutics and therapies.

## Introduction

Pancreatic diseases, including pancreatitis, pancreatic cancer, and type 1 diabetes mellitus (T1DM), not only exert an outsized adverse effect on human health because of marked morbidity and mortality, but impose a heavy societal burden worldwide (1). Pancreatitis is one of the most common gastrointestinal (GI) disorders seen in U.S. hospitals, ranking third after GI hemorrhage and gallbladder disease (1). Globally, the annual incidence rates of acute pancreatitis (AP) and chronic pancreatitis (CP) are 34 cases and 10 cases per 100,000 person-years, respectively (2). Around 80–85% of patients with AP develop interstitial edematous pancreatitis, with a mortality rate of <2%. Other patients might evolve into necrotizing pancreatitis of varying severity, and, in severe cases, where patients develop persistent organ failure, the mortality rate rises to 15–20% (3). In the cases of CP, there is an 8–9-fold increased risk of developing pancreatic cancer (4, 5). Pancreatic cancer is a common and fatal malignancy of the pancreas and is considered to be the third leading cause of death in the U.S. According to the “Cancer Facts & Figure 2020” issued by the American Cancer Society, the estimated number of deaths for pancreatic cancer is 7.8%, which is secondary to that of lung cancer (22.4%) and colorectal cancer (8.8%) (6). Notably, the incidence rate for pancreatic cancer increased by 1% per year from 2007 to 2016 and is projected to be the second biggest cancer killer in the U.S. by 2030 (6, 7). Another pancreatic disease, T1DM, does not directly manifest as gastrointestinal symptoms, but polyuria, polydipsia, and polyphagia (8). As one of the most common chronic disorders among children and youths (9, 10), the global incidence of T1DM has an average annual increase of 3–4% over the past three decades (11).

**Figure 1:**
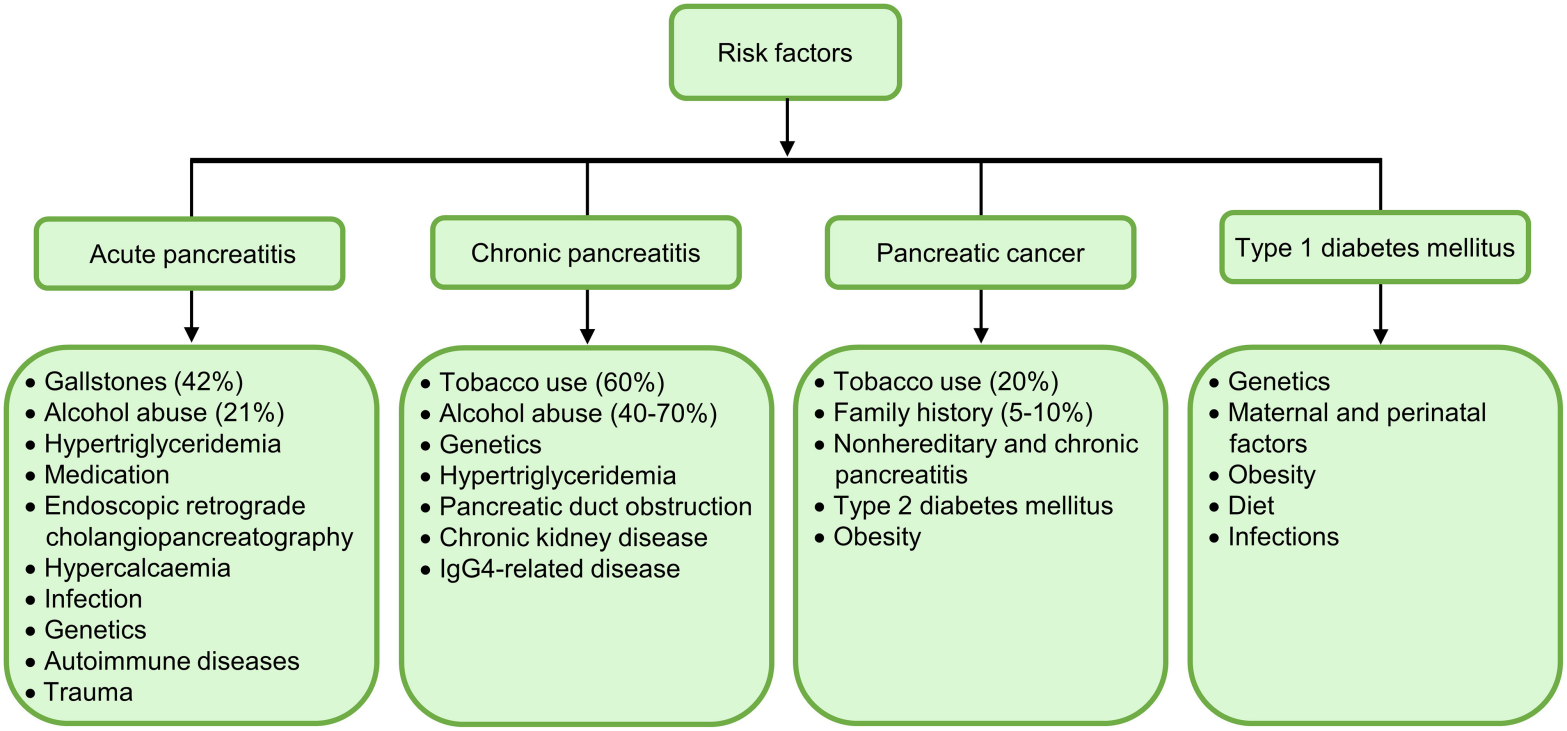
Summary of major aetiological risk factors for acute pancreatitis (AP), chronic pancreatitis (CP), pancreatic cancer, and type 1 diabetes mellitus (T1DM). Gallstones (42%) and alcohol abuse (21%) are the most frequent etiologies of AP; while tobacco use (60%) and alcohol abuse (40–70%) are the top aetiological factors of CP. Cigarette smoking (20%) and family history (5–10%) are dominant aetiological risk factors for pancreatic cancer. It is of note that alterations in oral microbiome predispose patients to pancreatic cancer. Genetics and environmental factors collectively contribute to the onset of T1DM, but other independent risk factors are unclear.

Despite the substantial improvement of our understanding of the cause, pathogenesis, and treatment of pancreatic diseases over the past decade (12–15), no curative treatment is yet available (16, 17). The World Health Organization (WHO) underscored the fundamental role that prevention plays before an episode of a disease in the global action plan for the prevention and control of non-communicable diseases 2013–2020, including GI disorders and diabetes (18). An aetiology-oriented strategy to disease prevention appears to be one of the most effective approaches for preventing and controlling pancreatic diseases (Figure 1). For example, identifying and eliminating environmental insults, such as smoking cessation and abstinence from alcohol, help prevent from pancreatitis and pancreatic cancer. It has been thought that T1DM is unpreventable (19, 20), but a recent study suggested that teplizumab, an Fc receptor-non-binding anti-CD3 monoclonal antibody, could delay or even prevent progression of this disease (21, 22). Therefore, apart from applying a disease prevention approach in managing these refractory diseases, novel therapeutics and curative treatments are desperately needed.

The human GI tract is home to trillions of microorganisms, including bacteria, archaea, fungi, viruses, and microeukaryotes, that are collectively known as the microbiome (23), forming a complex and unique ecosystem that influences human health and disease. Initially, our knowledge of the gut microbiome is largely reliant upon culture-dependent approaches. Breakthroughs in culture-independent approaches represented by high-throughput sequencing technologies, however, have further interrogated the intestinal microbial genomic blueprints and their functional potential in the past 16 years (24, 25). Likewise, the advent in mass spectrometry technology has facilitated the characterization and deciphering of gut microbial metabolite profiles (26). Accumulating evidence derived from metagenomics and metabolomics analyses suggests that gut microbiome composition and their metabolic activity are implicated in a multitude of conditions, including not only GI diseases (27, 28), but also extraintestinal disorders such as hepatic (29, 30), metabolic (31–40), respiratory (41–43), cardiovascular (44–47), neurologic (48–56), psychiatric (57–60), autoimmune (61–63), and oncologic components (64–69). There is no doubt that research into the gut microbiome and its role in human health and disease accompanied by tremendous technological advances in the past 16 years has taken center stage in biomedical science, and that most current microbiome research has revealed a close association between the gut microbiome and diseases, yet causative relationship has not been established in many cases. Given the bidirectionality of gut microbiome-disease interactions in human health, further research is needed in the future to dissect potential causality between them. In addition, gut dysbiosis is known to link to many disorders, and it thus seems feasible to manipulate the gut microbiome with products and treatments such as probiotics, prebiotics, synbiotics, postbiotics, and fecal microbiota transplantation (FMT) in clinical settings, irrespective of the stability and resilience of the intestinal microbiota (70–72).

This review summarizes existing evidence supporting the hypothesis that there is a close relationship between the gut microbiome and pancreatic diseases, and it further discusses the feasibility of preventing and controlling pancreatic diseases *via* microbiome-based therapeutics and therapies.

## The Gut Microbiome and Healthy Pancreas

**Figure 2:**
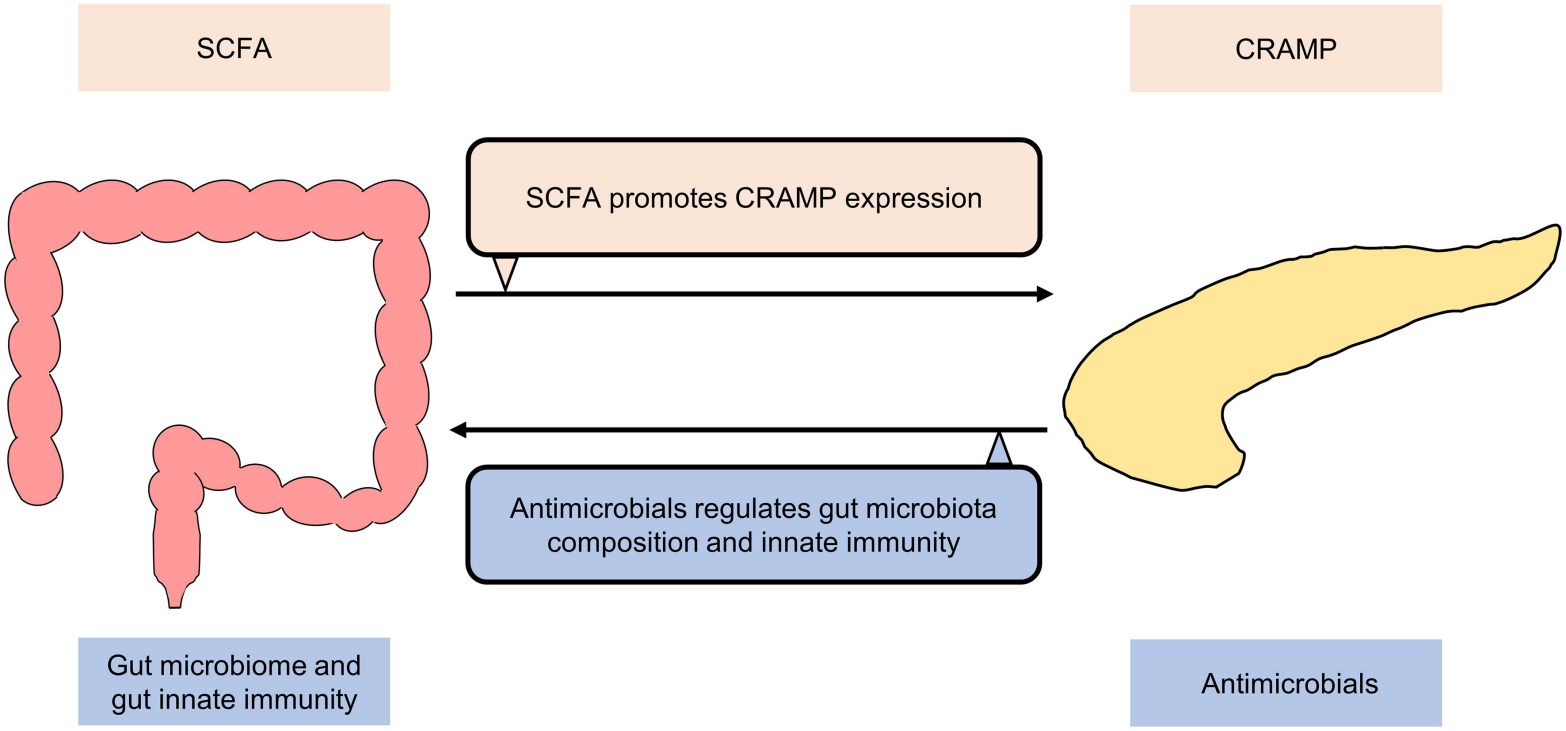
Bidirectional communication between gut and pancreas under normal conditions. Cathelicidin-related antimicrobial peptide (CRAMP) production by insulin-secreting beta-cells is controlled by short-chain fatty acids (SCFA) produced by the gut microbiota. ORAI calcium release activated calcium modulator 1 (Orai1) produced by pancreatic acinar cells mediates the secretion of antimicrobials, which shapes the gut microbiome and regulates gut innate immunity.

The fact that *Helicobacter pylori*-related disease (73–75) and recurrent *Clostridium difficile*-associated disease (76–78) are closely related to the gut microbiome has pushed the research into the functional interplay between the gut microbiome and human health/disease to a climax. An active role of the gut microbiome in influencing extraintestinal diseases has been increasingly recognized. More recently, research focus has thus shifted to investigate the relationship between the gut microbiome and extraintestinal manifestations like pancreatic diseases (79). Anatomically, the pancreas is located behind the stomach, where it connects the duodenum through the pancreatic duct and communicates with the liver through the common bile duct, forming a subtle gut-pancreas-liver axis for bidirectional communications. Experimental evidence also supports the existence of direct pancreas-gut microbiome interactions (Figure 2). For example, the production of cathelicidin-related antimicrobial peptide (CRAMP) by insulin-secreting beta-cells was controlled by gut microbiota-originated short-chain fatty acids (SCFA), highlighting a direct role of the gut microbiota in shaping the pancreatic immune microenvironment (80). On the other hand, Ahuja and colleagues demonstrated that ORAI calcium release-activated calcium modulator 1 (Orai1) produced by pancreatic acinar cells mediated secretion of antimicrobials that shaped the intestinal microbiota and intestinal immunity (81). These two seminal studies shed light on the existence of a gut-pancreatic axis, which warrants further preclinical and clinical investigations.

In addition, one caveat is that both normal and diseased individuals harbor a pancreatic microbiome (82), which might engage in disease initiation and progression (83–85). The landmark study by Pushalkar and colleagues discovered a link between pancreatic tumorigenesis and gut-originated intrapancreatic bacteria (84). It is notable that not only bacteria were the culprits, but also fungi. Gut-residing fungi that migrated from the intestine to pancreas might facilitate the progression of pancreatic ductal adenocarcinoma (PDAC) by driving the C3 cascade through activating mannose-binding lectin (85).

These studies challenged the conventional belief over the past decades that pancreas is a sterile organ and supported the presence of a pancreatic microbiome. Moreover, the results of various studies using traditional culture methods, real-time quantitative polymerase chain reaction (qPCR), 16S rRNA and shotgun metagenomics sequencing technologies consistently showed that the gut microbiota could migrate between the intestine and pancreas though the route of migration remains elusive. Three translocation pathways have been proposed: pancreatic duct reflux route, mesenteric venous drainage, and mesenteric lymphatic drainage (79). In-depth knowledge of the disease-associated microbial signatures, the exact mechanisms and trajectories of microbial migration, and the specificity of microbes capable of translocating through these routes may offer novel therapeutic targets and strategies.

Collectively, these works suggest that a gut-pancreatic axis may exist, which plays a role in determining the onset and severity of pancreatic diseases. Untangling this subtle yet intimate communication loop requires further preclinical and clinical studies.

## The Gut Microbiome and Pancreatic Diseases

### The Gut Microbiome in Pancreatitis

#### Acute Pancreatitis

A common inflammatory disorder of the pancreas is AP, in which pancreatic enzymes are activated locally due to a variety of etiologies, causing autodigestion, edema, hemorrhage, and even necrosis of pancreatic tissue, as well as dysfunction of remote organs and systems (86). Although the pathophysiology of AP has been well-described (87), none of these has pointed to the attribution to the gut microbiome. However, patients with AP, especially severe AP (SAP), are often accompanied by intestinal barrier dysfunction and subsequent bacterial translocation into the pancreas and/or peripheral blood (88, 89), and since the intestinal microbiome plays a vital role in maintaining intestinal epithelial barrier integrity and shaping intestinal mucosal immune system (90–92), the importance of the gut microbiome in the aetiology of AP should not be neglected.

It was not until recently that researchers began to apply high-throughput sequencing technologies in deciphering the role of gut dysbiosis in AP. For instance, gut dysbiosis and decreases in the expression of antimicrobial peptides in Paneth cells were observed in rats suffering from acute necrotizing pancreatitis induced by hypertriglyceridemia (93). 16S rRNA-based gut microbiota analysis revealed that patients had fewer commensal beneficial bacteria (such as *Prevotella* and *Faecalibacterium*) and more pathogenic bacteria (such as *Escherichia-Shigella* and *Enterococcus*) compared with healthy subjects (94). Moreover, a study found that patients with varying severity of AP had distinct gut microbiota signatures, characterized by more *Bacteroides, Escherichis-Shigella*, and *Enterococcus* in mild AP (MAP), moderately SAP (MSAP), and SAP, respectively (95). Similarly, another study reported that the intestinal microbiome signature in patients with SAP was distinct from those suffering from MAP and MSAP, characterized by a reduction in commensal bacteria such as *Bacteroides, Alloprevotella*, and *Blautia* (94).

Despite the investigators have observed changes in intestinal microbiome in the setting of AP, few studies exist that have explored the effect of intestinal microbiome on AP. A few exceptions do exist, a seminal experimental study showed that antibiotic-treated mice and germ-free (GF) mice exhibited attenuated pancreatic injury after AP induction, and subsequent FMT worsened the severity of AP, demonstrating that the gut microbiota was a mediator in AP (94). This notion is underpinned by another recently published study where the authors reported that the gut microbiota and the NLRP3 inflammasome acted together to exacerbate the severity of AP (96). Specific intestinal species even appeared to be critical for the pathogenesis. For example, the gut commensal species, *Escherichia coli*, exacerbated acute necrotizing pancreatitis through targeting intestinal epithelial cells (97). These pioneering studies strongly support that the gut microbiota and dysbiosis are associated with the severity of AP.

#### Chronic Pancreatitis

Another common pancreatic disorder is CP. It is a progressive fibroinflammatory condition characterized by gradual replacement of pancreatic secretory parenchyma by fibrous tissues, resulting in endocrine and exocrine dysfunction (98). Similar to AP, the pathogenesis in CP might be associated with the gut microbiome (99–101). A seminal study systematically investigated the fecal microbiomes of patients with CP by 16S rRNA gene sequencing (102). The 16S rRNA microbiota in patients with CP had diminished gut microbial diversity and richness, and the dysbiosis was accompanied by alterations in the taxonomic microbiota profiles (102). A similar outcome was also observed for the first time in mice with CP where authors showed that CP mice had significantly reduced bacterial species richness and diversity (103). Specifically, the abundance of *Bacteroides* and *Alloprevotella* genera increased, while the abundance of *Lachnospiraceae_NK4A136, Ruminiclostridium*, and *Roseburia* decreased (103). A recent study also demonstrated a distinct difference in gut microbiota between CP mice and control mice (104). However, to our knowledge, no study to date has yet examined the role of intestinal microbiome on CP in humans or animals, which is worthy of future investigation.

Altogether, research on the gut microbiome of individuals with pancreatitis is still in its infancy, but current evidence does implicate that the host gut microbiome and pancreatitis are closely linked. Applications of GF mouse models and metagenomic sequencing approaches rather than relying on biomarker sequencing will likely provide a better insight into the intricate relationship between the gut microbiome and pancreatitis.

### The Gut Microbiome in Type 1 Diabetes Mellitus

T1DM is an autoimmune disorder, characterized by T cell-mediated destruction of insulin-producing beta cells in the pancreas, resulting in a reliance on exogenous insulin throughout life (105). Genetic variation is a well-established risk factor for T1DM as more than 50 diverse genetic loci have been identified (106), many of which are located in the human leukocyte antigen (HLA) region (107). Apart from genetic predisposition to T1DM pathogenesis, environmental factors also play an integral role (108). In fact, genetic susceptibility and environmental events, conspire together to provide fertile ground for the initiation and progression of T1DM (109), and since both environmental factors (e.g., dietary habits) (110–113) and genetic risks (114–116) profoundly affect the human gut microbiome, it would be of interest to find out the role of the gut microbiome in T1DM.

In fact, the association between the gut microbiome and T1DM has been implicated in previous studies (117–122). 16S rRNA pyrosequencing analysis of fecal microbiota of children with and without beta-cell autoimmunity showed that children with autoimmunity had fewer lactate-producers, butyrate-producers, *Bifidobacterium adolescentis*, and *Bifidobacterium pseudocatenulatum* but more *Bacteroides* (123). Another study compared the gut microbiota of children with T1DM, maturity-onset diabetes of the young 2 (MODY2), and in healthy state in a case-control study by 16S rRNA pyrosequencing, and the results showed that children with T1DM had a significantly lower gut microbiota diversity; significantly more *Bacteroides, Ruminococcus, Veillonella, Blautia*, and *Streptococcus genera*; and significantly fewer *Bifidobacterium, Roseburia, Faecalibacterium*, and *Lachnospira* (124). Gut dysbiosis was observed in children aged 1–5 years with new-onset T1DM (125). Whole metagenome sequencing uncovered an increase in lipopolysaccharides-producing bacteria and a decrease in SCFA-producing bacteria in women with T1DM across pregnancy compared with healthy individuals as controls (126). Remarkably, the gut mycobiome signature is beginning to intrigue humans, as recently demonstrated in a pilot study assessing the gut mycobiome in adult patients with T1DM (127).

Indeed, the gut microbiome appears to be a principal driving force of the onset of T1DM, particularly in subjects genetically disposed to the disease. The hallmark study of Wen and colleagues first reported that GF but not specific pathogen-free non-obese diabetic (NOD) mice devoid of MyD88, a key intracellular component of multiple Toll-like receptor-mediated signaling pathways, did develop robust diabetes, and colonization of a defined microbial consortium could mitigate diabetes (128). A longitudinal study followed the serum conversion pattern of autoantibody of 33 HLA-matched infants from birth to 3 years of age (129). Four of the 11 seroconverted infants developed T1DM, accompanied by drastic reduction in intestinal microbiome diversity though no obvious change was observed in their major gut metabolites (129). On the other hand, The Environmental Determinants of Diabetes in the Young (TEDDY) study analyzed stool samples of 783 children collected monthly during their first 3 months of age until the clinical endpoint (130). The study found that early infant microbiome was dynamic and highly individualized both at the taxonomic and functional levels, and the colonic SCFA might influence early-onset T1DM (130). The gut microbiome not only drives T1DM development, but also has an impact on cognitive functions. For examples, depletion of acetate producing bacteria, caused by vancomycin exposure, resulted in cognitive impairment in T1DM mice (131). Moreover, gender bias in autoimmunity including T1DM was found to be influenced by the host gut microbiota, and the sex differences in the gut microbiome could drive hormone-dependent regulation of autoimmunity (132, 133). Thus, experimental and clinical evidence implicated that alterations in the gut microbiome and/or metabolome occurred prior to or even played an active role in driving the onset of T1DM in genetically susceptible infants, though the exact mechanisms are largely unclear. Yet, a previous study found that lipopolysaccharide from *Bacteroides dorei* might increase the risk of autoimmunity (134). Moreover, only very few studies applied metaproteomics in investigating the functional interactions between the host microbiota and T1DM disease risk (135).

Thus, it will be necessary to conduct more mechanistic studies to confirm the causal role of gut microbiome in the onset of T1DM not only by 16S rRNA gene sequencing, but also in combination with metagenomics, metabolomics, and metaproteomics approaches to decipher the functionality of the gut microbiome in T1DM.

### The Gut Microbiome in Pancreatic Cancer

Pancreatic cancer refers to tumors that start in the cells of the pancreas, including exocrine tumors. Generally, it is classified into PDAC (about 95% of pancreatic cancers) and endocrine tumors (about 5% of pancreatic cancers) (136). In PDAC, alterations in gut microbiota were observed both in humans and animals. For example, genetically engineered PDAC mice exhibited gut dysbiosis characterized with the predominance of Proteobacterial and Firmicutes as well as elevated serum polyamine metabolism (137). A previous study found that the 16S rRNA fecal microbiota of patients with pancreatic carcinoma (*n* = 85) exhibited significantly diminished alpha-diversity compared with matched healthy controls (*n* = 57), accompanied by increases in certain pathogens and lipopolysaccharides-producing bacteria, and decreases in probiotics and butyrate-producing bacteria (138). Patients with PDAC had an increased phylum Proteobacteria (such as Gammaproteobacteria) and a decreased phylum Firmicutes (such as butyrate-producing bacteria, including *Eubacterium rectale, Faecalibacterium prausnitzii*, and *Roseburia intestinalis*) when compared with healthy controls (139). Additionally, several recent review articles have underscored the importance of the gut microbiome in the development and progression of PDAC (140–142).

Evidence is also beginning to show that the gut microbiome is involved in pancreatic oncogenesis or tumor suppression (143). Thomas and colleagues demonstrated that the intestinal microbiome could accelerate pancreatic carcinogenesis through a long-distance mechanism in preclinical models (144). Ablation of the gut microbiome by oral antibiotics could diminish pancreatic malignancies burden through increasing infiltration of interferon gamma-producing T cells and reducing interleukin 17A and interleukin 10-producing T cells, further supporting the notion that the gut microbiome might promote the pathogenesis in pancreatic cancer (67).

Recent studies found that the pancreatic tissue harbors microorganisms under both normal and pathological conditions (82). A unique microbial signature has also been observed in patients with pancreatic cyst fluid, characterized by an enrichment of *Bacteroides* spp., *Escherichia/Shigella* spp., and *Acidaminococcus* spp. (145). The fact that the pancreas harbors microorganisms raises the question of whether the pancreatic microbiome, in addition to the gut microbiome, is tied to the disease process. Geller and colleagues showed by qPCR, 16S rRNA fluorescence in situ hybridization analysis, and immunohistochemistry that intratumor samples of pancreatic cancer patients contained more bacteria than matched tissues in control subjects (83). The intratumor microbiota of these human PDAC samples was dominated by Gammaproteobacteria, which conferred gemcitabine resistance in 14 of 15 of the human PDAC tumors (83). Collectively, these data showed that pancreatic tumor tissues contained bacteria that rendered resistance to gemcitabine and thereby affected the efficacy of cancer therapies (83). The study convincingly confirmed the role of intratumor bacteria in promoting gemcitabine resistance in pancreatic cancer.

More intriguingly, the observations of several recent studies suggested that the gut microbiome was in tight partnership with the intratumoral microbiome collectively contributing to carcinogenesis in the tumor microenvironment, implicating that there was gut-tumor microbial crosstalk and that such interactions could affect tumor outcome (84, 85). The tumor microbiome in PDAC patients also varied with tumor tempo; the 16S rRNA-tumor microbiome of patients of long-term survival (LTS) exhibited a higher alpha-diversity and more *Pseudoxanthomonas, Saccharopolyspora, Streptomyces*, and *Bacillus clausii* than patients of short-term survival (STS) (146). Mechanistically, the tumor microbiome shaped the antitumor immune responses through CD8^+^ T cell recruitment and activation (146). Furthermore, human gut microbiome was absent from normal tissues adjacent to tumor tissues, but it represented 25% of human tumor microbiome in matched tumor samples (146). The FMT of fecal samples of LTS but not STS or healthy donors significantly reduced tumor growth in antibiotics-fed mice with orthotopic syngeneic tumors, and the antitumor effect was attenuated by antibiotic treatment (146). These results suggested that the gut microbiota of pancreatic cancer patients had the capacity to migrate and colonize pancreatic tumor tissues and that the gut and/or tumor bacteria from LTS patients could confer a protective effect against tumor growth. It would thus be of interest to further elucidate the significance of gut-tumor microbial crosstalk in progression of pancreatic cancer. Moreover, since the oral microbiome is closely related to the gut microbiome, it is also worthy investigating if the oral microbiome is another driver for the progression of pancreatic cancer (147–149).

**Figure 3:**
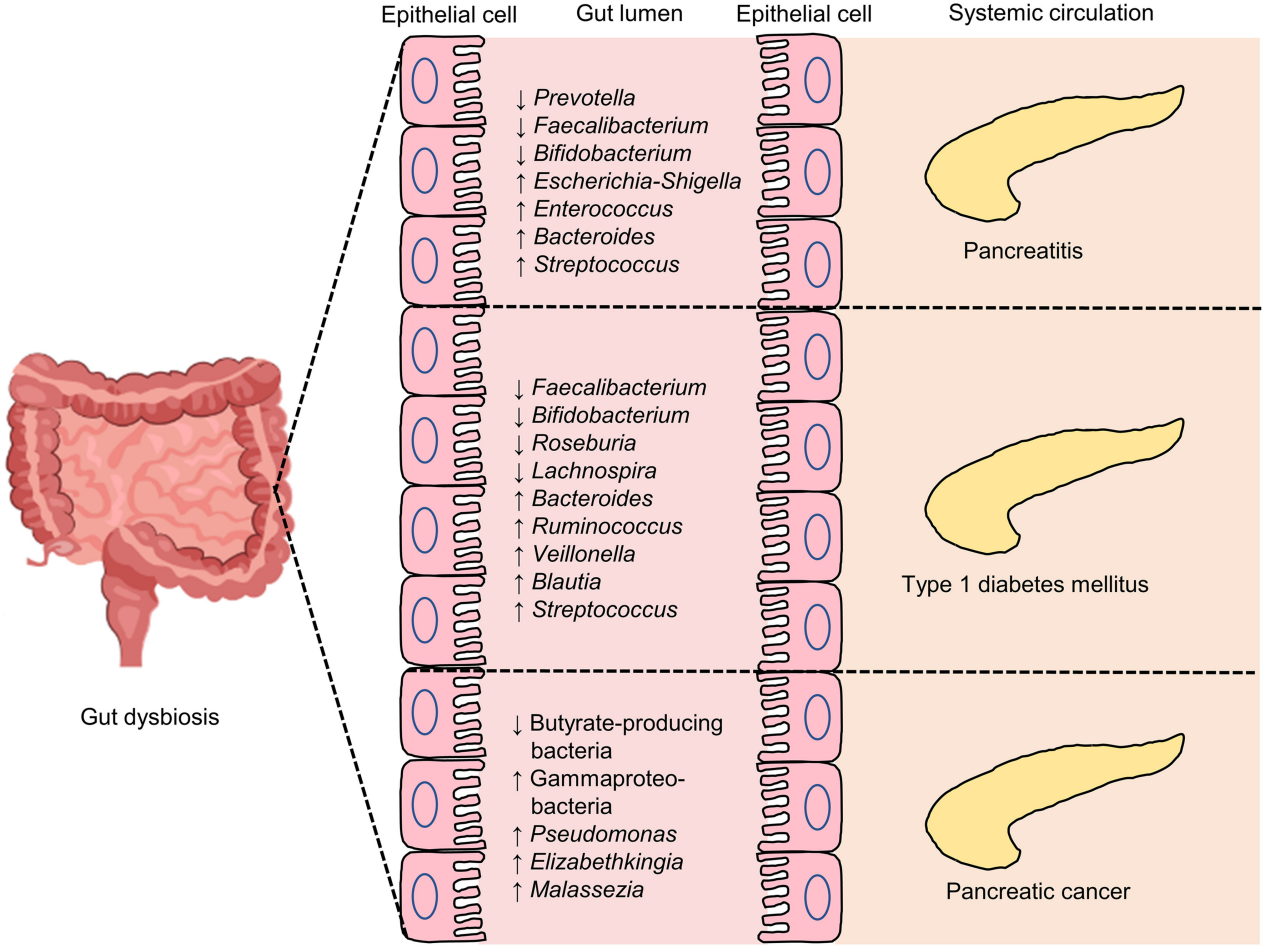
Some of the reported intestinal microbial genic features in pancreatic diseases, including pancreatitis, type 1 diabetes mellitus (T1DM) and pancreatic cancer. An overview of selected key gut microbiome features related to pancreatic diseases. ↓, lower abundance in pancreatic diseases when compared with control; ↑, higher levels in pancreatic diseases when compared with control.

In summary, a subtle yet intimate nexus loop exists between the gut microbiome and pancreatic diseases, including pancreatitis, pancreatic cancer, and T1DM (Figure 3). Gut dysbiosis has been consistently reported in patients of pancreatic diseases; however, causal relationships between the gut microbiome and pancreatic disorders, as well as the mechanism of gut microbiome in contributing to the onset, progression, and pathogenesis of this spectrum of diseases, merit further elucidation. The expansion of our knowledge in the field will help identify novel treatment targets and deepen our insights into the development of personalized therapies.

## Gut Microbiome-Target Therapies

**Figure 4:**
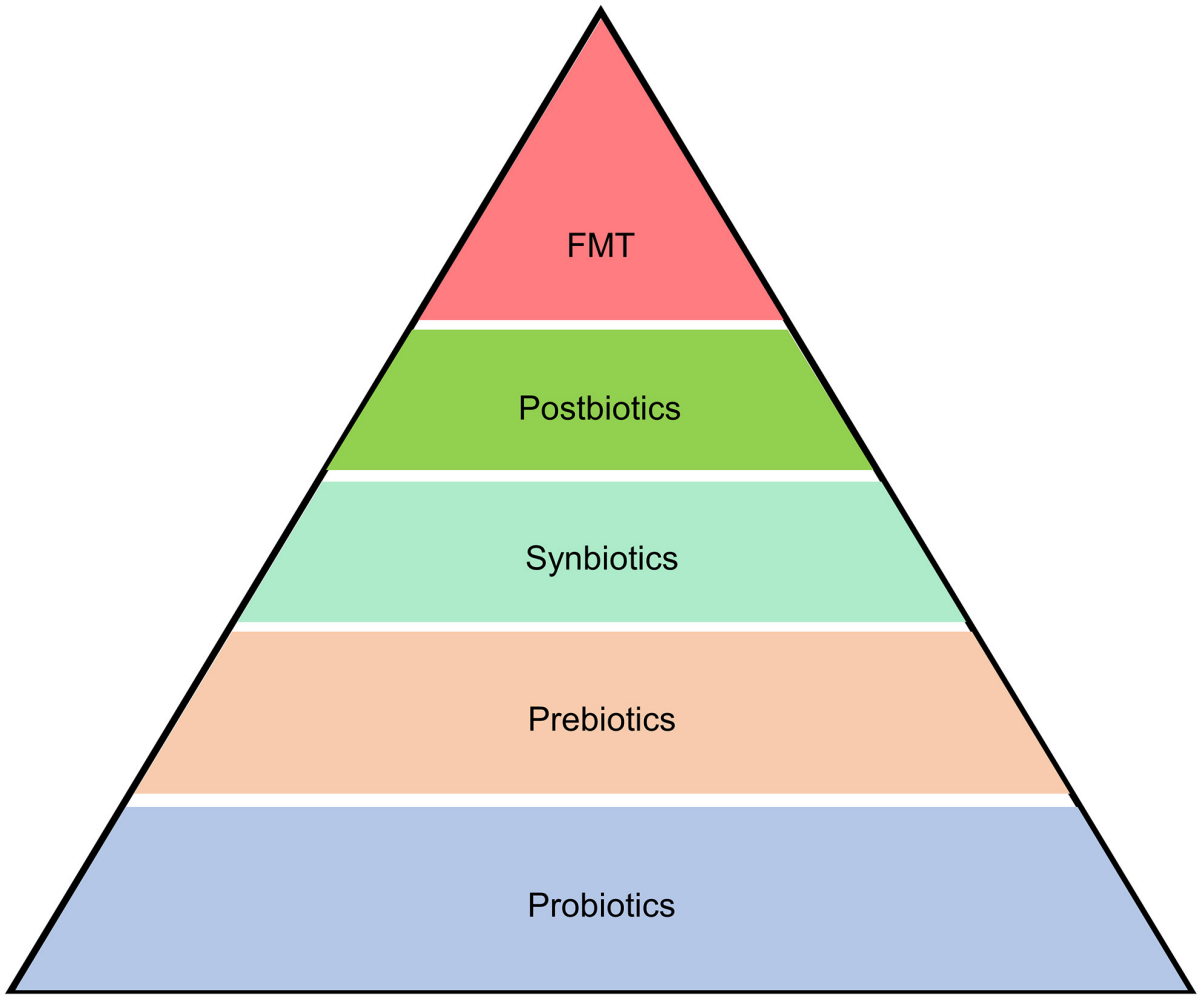
Microbiome-based therapeutics and therapies for pancreatic diseases. Strategies to alleviate pancreatic diseases by modulating the gut microbiome include application of products and treatments like probiotics, prebiotics, synbiotics, postbiotics, and fecal microbiota transplantation (FMT).

Given the close link between the gut microbiome and pancreatic disorders, it may be feasible to ameliorate pancreatic diseases by modulating the gut microbiome *via* application of products and treatments like probiotics (150), prebiotics (151), synbiotics (152), postbiotics (153), and FMT (154) (Figure 4). A better understanding of the causal relationship in gut microbiome-pancreatic disease interactions will improve the precision of novel target therapies.

### Probiotics

Probiotics are defined as “live microorganisms that, when administered in adequate amounts, confer a health benefit on the host,” according to the International Scientific Association for Probiotics and Prebiotics (ISAPP) consensus in 2013 (150). The role of probiotics in human health and disease has received increasing attention, especially in the prevention and treatment of acute gastroenteritis (155), *Clostridium difficile*-associated diarrhea (156–158), irritable bowel syndrome (159, 160), neonatal sepsis (161), and acute respiratory infection (162), which have shown beneficial clinical efficacy. Although less studied, clinical evidence also shows beneficial effects of administering probiotics in pancreatic-related disorders.

#### Pancreatitis and Probiotics

A number of preclinical studies have reported beneficial effects of probiotic application in mitigating AP (Table 1). For instance, ingesting *Lactobacillus plantarum* for 4 days before and after induction of AP could reduce microbial translocation in experimental pancreatitis (163). Consuming a microbial consortium comprising *Streptococcus thermophilus, Lactobacillus acidophillus, Bifidobacterium lactis* was capable of reducing the severity of experimental AP (164). A similar outcome was observed in rats that received Ecologic 641 probiotic formulations 5 days prior to the induction of AP (165). Modification of gut microbiota *via* administration of Ecologic 641 probiotic formulations reduced bacterial translocation, morbidity, and mortality in the course of experimental AP (166). In two independent studies, the authors observed that *Saccharomyces boulardii* could diminish bacterial infections and ameliorate pancreatitis (167, 168). A systematic review and meta-analysis strengthened the findings of the aforementioned studies by showing that probiotics did exhibit efficacy in animal models of AP (169). Although the probiotic product, Ecologic 641 probiotic formulations, has been widely promoted as beneficial for AP, a rat experimental model aiming to investigate the association between probiotic prophylaxis (by feeding Ecologic 641 probiotic formulations) and enteral nutrition with AP mortality did not find significant differences between groups in terms of histological severity of pancreatitis, degree of discomfort, weight loss, histological examination of small bowel and bacterial translocation, suggesting probiotic application was ineffectual nor harmful to AP-induced rats (170). Another study found that *Clostridium butyricum* and its major metabolite, butyrate, could reduce intestinal injury possibly by altering the functions of the intestinal mucosal barrier (171).

**Table 1:** Summary of preclinical studies investigating the effects of probiotic application in acute pancreatitis (AP).

**Probiotic species/product**	**Probiotic dose**	**Time of probiotic application**	**Main observations**	**Reference(s)**
*Lactobacillus plantarum*	2.5–5 ×10^9^ CFU/d	4 d before and after induction of AP	Reduced microbial translocation in experimental pancreatitis	(163)
*Streptococcus thermophilus, Lactobacillus acidophillus*, and *Bifidobacterium lactis*	2.4 ×10^9^ CFU/d	5 d after induction of AP	Reduced the severity of AP	(164)
Ecologic 641	5 ×10^9^ CFU/d	5 d before induction of AP	Ameliorated the severity of AP *via* reducing oxidative stress-induced injury	(165)
Ecologic 641	5–10 ×10^9^ CFU/d	5 d before and 7 d after induction of AP	Reduced bacterial translocation, morbidity, and mortality	(166)
*Saccharomyces boulardii*	50 mg/kg/d	6 h and 30 h after induction of AP	Diminished bacterial infections and offer health benefits	(167)
*Saccharomyces boulardii*	50 mg/kg/d	6 h and 24 h after induction of AP	Reduced bacterial translocation	(168)
Ecologic 641	5 ×10^9^ CFU/d	4 d before and 6 d after induction of AP	No differences in histological severity of pancreatitis and bacterial translocation between groups	(170)
*Clostridium butyricum*	10^9^ CFU/d	11 d before induction of AP	Reduced intestinal injury	(171)

*1. “Ecologic 641” was a probiotic mix comprised six strains of freeze-dried, viable bacteria: Lactobacillus acidophilus, Lactobacillus casei, Lactobacillus salivarius, Lactococcus lactis, Bifidobacterium bifidum, and Bifidobacterium lactis (previously classified as Bifidobacterium infantis), plus cornstarch and maltodextrins*.

It was not until 2002 that probiotics began to be used in clinical trials (Table 2). In Oláh et al. (172), 45 patients with AP were randomly assigned to receive either live or inactivated *Lactobacillus plantarum* (that is, postbiotics) with oat fiber for 7 days by nasojejunal tube. Significant differences were observed in the severity of infective pancreatic necrosis and abscesses between the two groups, but not the mean length of hospital stay (172). Similarly, *Lactobacillus plantarum* supplementation in patients with AP attenuated the severity of disease, improved intestinal permeability and clinical outcomes (173). Although beneficial effects of ingesting probiotics have been observed in some clinical studies in AP, other studies yielded neutral or even negative outcomes. For example, in a multicenter randomized, double-blind, placebo-controlled trial, 298 patients with SAP were randomly assigned to receive either Ecologic 641 probiotic formulations (*n* = 153) or placebo (*n* = 145), infectious complications occurred in 46 (30%) probiotic-receivers and 41 (28%) placebo-receivers, and the mortality rate of patients was higher in the probiotic group (24 patients; 16%) compared with (9 patients; 6%) the placebo group, suggesting that probiotic consumption did not reduce the risk of infectious complications but was associated with an increased risk of mortality (174). Probiotic supplementation did not affect gut integrity, infectious complications, mortality, and hospital stay in another double-blind randomized controlled trial published in 2011 (175). Furthermore, two meta-analyses reported that probiotics intake was neither beneficial nor harmful to patients with SAP (176, 177). A recent Cochrane review that included 84 randomized controlled trials found that, probiotics intake was not associated with the overall mortality in AP (178). Intriguingly, probiotic preparations consisting of *Bifdobacterium longum, Lactobacillus acidophilus*, and *Enterococcus faecalis* showed a curative effect in patients with SAP by lowering the levels of pro-inflammatory cytokines, restoring the gastrointestinal function sooner, decreasing complications such as infection, and shortening of hospital stay in patients with SAP (179). The consumption of a mixed probiotic preparation containing *Bacillus subtilis* and *Enterococcus faecium* reduced the percentage of pancreatic sepsis, multiple organ dysfunction syndrome, and mortality (180). Furthermore, the intake of the same probiotic mix reduced the length of hospital stay of patients with MAP, although no statistical difference was seen in recurrent abdominal pain between the probiotic and placebo group (181).

**Table 2:** Summary of clinical trials investigating the efficacy of probiotic application in pancreatitis.

**Probiotic group**	**Control group**	**Treatment frequency; time**	**Clinical trial design**	**Main observations**	**Reference(s)**
10^9^ CFU/d *Lactobacillus plantarum*+ oat fiber	Inactivated *Lactobacill-us plantarum*+ oat fiber	Twice daily; 7 d	Randomized, double-blind	Reduced pancreatic sepsis and the number of surgical interventions	(172)
10^10^ CFU/d *Lactobacillus plantarum*	Normal saline	Once daily; 7 d	Randomized, single-blind	Attenuated disease severity, improved intestinal permeability and clinical outcomes	(173)
10^10^ CFU/d Ecologic 641	Placebo	Twice daily; 28 d	Multicenter, randomized, double-blind, placebo-controlled	Did not reduce risk of infectious complications, increased risk of death	(174)
10^10^ CFU/d Probiotic sachet	Placebo	Once daily; 7 d	Randomized, double-blind, placebo-controlled	No effect on intestinal permeability or endotoxemia	(175)
2.1 ×10^10^ CFU/d *Bifidobacterium* triple viable capsules	Water	Twice daily; 14 d	Randomized	Reduced the level of proinflammatory cytokines, restored gastrointestinal function earlier, reduced the occurrence of complications	(179)
3 ×10^9^ CFU/d Live combined *Bacillus subtilis* and *Enterococcus faecium* enteric-coated capsules	Water	Third daily; 14 d	Randomized, double-blind	Reduced pancreatic sepsis, multiple organ dysfunction syndrome, and mortality	(180)
– Live combined *Bacillus subtilis* and *Enterococcus faecium* enteric-coated capsules	Placebo	–; 30 d	Randomized, double-blind, placebo-controlled	Shortened the length of hospital stay; no statistical difference in recurrent abdominal pain	(181)

*1. “Ecologic 641” was a probiotic mix comprised six strains of freeze-dried, viable bacteria: Lactobacillus acidophilus, Lactobacillus casei, Lactobacillus salivarius, Lactococcus lactis, Bifidobacterium bifidum, and Bifidobacterium lactis (previously classified as Bifidobacterium infantis), plus cornstarch and maltodextrins*.

*2. The “Probiotic sachet” contained Lactobacillus acidophilus, Bifidobacterium longus, Bifidobacterium bifidum, and Bifidobacterium infantalis in addition to 25 mg of fructooligosaccharide*.

*3. “Bifidobacterium triple viable capsules” contained Bifdobacterium longum, Lactobacillus acidophilus, and Enterococcus faecalis*.

*4. –, not available*.

Overall, there are discrepant results regarding the effects of probiotics on AP. Larger scale studies would be necessary to clarify whether probiotics intake could improve AP and if there are strain-specific effects in alleviation of AP-associated symptoms. It is noteworthy that there is scarce data on the effect of probiotics on CP in preclinical and clinical trials; thus, whether probiotic treatment has any clinical efficacy in CP remains to be confirmed.

##### Probiotic Mechanisms of Action

**Figure 5:**
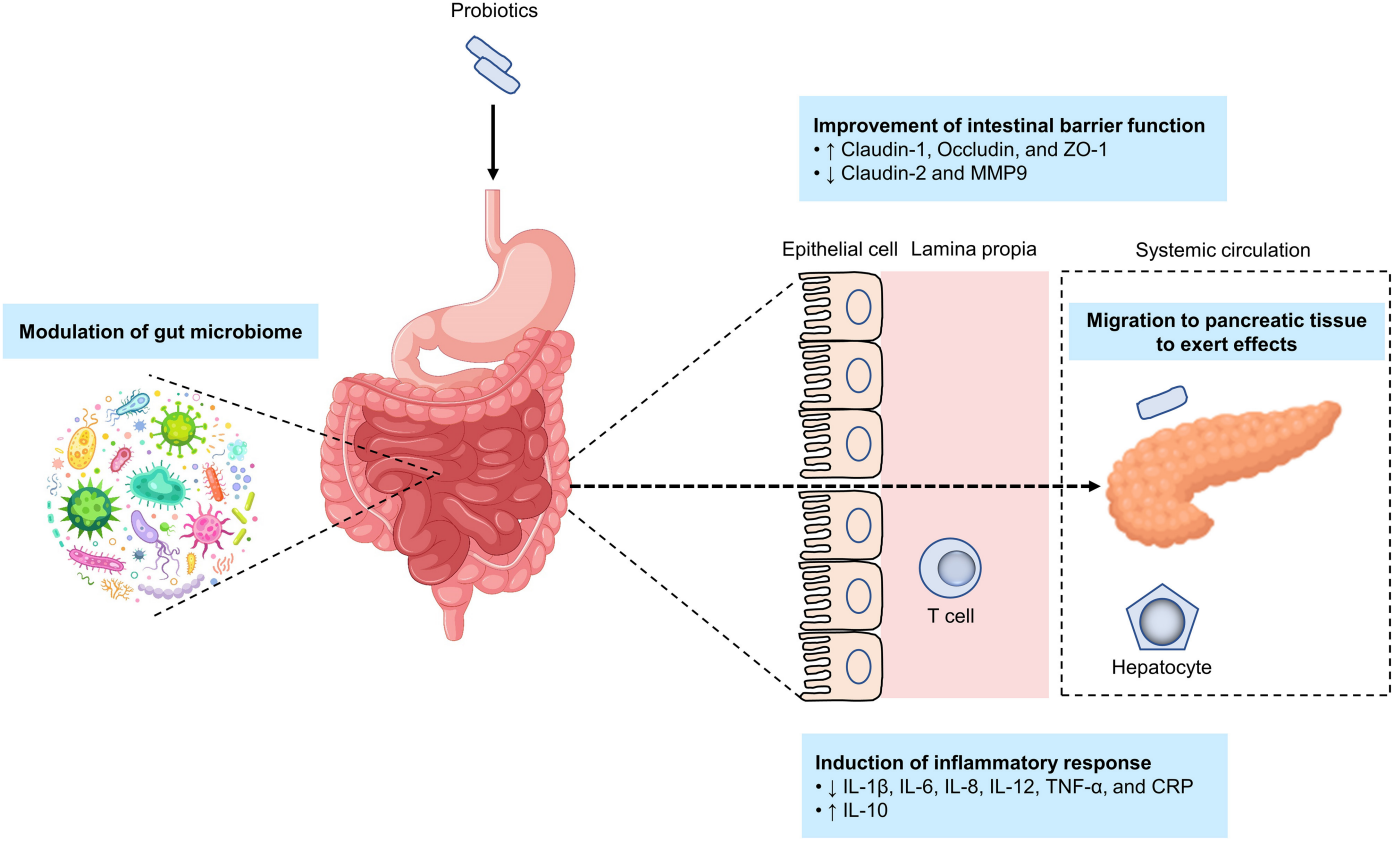
Potential mechanisms by which probiotics alleviate acute pancreatitis. The putative mechanisms by which probiotics alleviate acute pancreatitis include improving the intestinal barrier function, inducting inflammatory responses, modulating the gut microbiome, and targeting pancreatic tissue. ↑, indicates an increase; ↓, indicates a decrease.

The available literature reveals that the mechanisms of probiotic effects in AP are exerted *via* improving the intestinal barrier function, inducting inflammatory responses, and modulating the gut microbiome (Figure 5).

Intestinal barrier dysfunction is closely linked to the course of AP (89). Bacterial translocation is triggered by an imbalance in intestinal barrier function, especially bacterial migration from the small intestine into the pancreatic tissue and/or in peripheral blood is a major cause of infection in the pancreas or peripancreatic tissue (88, 182). Probiotics may act by improving intestinal barrier function that in turn prevents bacterial translocation. Oral gavage of Ecologic 641 probiotic formulations once daily for 2 days to mice prior to induction of AP by intraperitoneal injections with cerulein could prevent intestinal barrier dysfunction in the late phase of AP; however, such effect was not seen if probiotics were administered after induction of AP, indicating that the efficacy of probiotics was related with the timing of probiotic application (183). In a randomized, placebo-controlled, multicenter trial, probiotic application showed a capacity for preventing bacterial translocation (184). Another study found that the probiotic mechanism of improvement of intestinal barrier function was *via* increasing tight-junction proteins (including claudin-1, occludin, and ZO-1) while reducing claudin-2 and MMP9 (171). It is worth noting that the metabolites of probiotics, also known as postbiotics similarly have the effect of improving intestinal barrier function and thus preventing bacterial translocation (185–187).

As discussed earlier, bacteria and fungi are able to migrate from the gut into the pancreatic tissue, where they are responsible for the course of disease (84, 85). Translocation of a gut pathobiont, *Enterococcus gallinarum*, to the liver and other systemic tissues triggered autoimmune responses in genetically predisposed hosts provides a prime example (188). Moreover, the dissemination of *Escherichia coli* from primary colorectal cancer (CRC) *via* the gut vascular barrier allowed the bacteria to migrate to the liver to form a premetastatic niche, paving a way for CRC to metastasize to the liver in advance (189). Despite the aforementioned two studies are beyond the scope of this review, they have provided compelling evidence of dissemination of gut-originated bacteria in relation to onset and development of diseases. In our laboratory, we found that *Lactobacillus rhamnosus* Probio-M9 could transmit from the gut to mammary tissue to alleviate symptoms of *Staphylococcus aureus*-induced mastitis in rats. Here, we hypothesize that the mechanism of action of probiotics resembles that seen in several examples mentioned above, albeit we do not know whether probiotics will promote or alleviate the development of disease (Figure 5).

Another probiotic mechanism is *via* reducing inflammatory responses in pancreatitis. Ingesting probiotics could lower proinflammatory cytokines, such as interleukin (IL)-8, tumor necrosis factor (TNF)-α, and C-reactive protein (179). Meanwhile, the species *Clostridium butyricum* has been shown to suppress IL-6, IL-12, IL-1β, and TNF-α production (171), while another study found that probiotics intake could reduce TNF-α and IL-6 expression and enhanced IL-10 expression in SAP (180).

Finally, probiotics might serve as a gut microbiota modifier, regulating the gut function and homeostasis (190), particularly in the presence of pancreas-gut microbiota cross-talk and interactions (143). Notably, few studies have investigated the effects and mechanisms of action of probiotics on the gut microbiome in AP. Large-scale animal models and high-quality clinical trials are therefore needed to determine the interactions between probiotics, host gut microbiome, and AP development and pathogenesis.

#### T1DM and Probiotics

The NOD mouse model is one of the most important animal models in T1DM research (191). A myriad of evidence regarding the role of probiotics in T1DM stems from NOD mouse models and human studies (Table 3). Female NOD mice receiving the probiotic mix, VSL#3, orally could prevent spontaneous autoimmune diabetes *via* an IL-10-dependent mechanism and gut microbiota modulation (192, 193). A previous study in NOD mice showed that *Lactococcus lactis* could serve as a potential immunotherapeutics for autoimmune T1DM (194). As one of the examples, oral administration of *Lactococcus lactis* secreting the beta-cell antigen glutamic acid decarboxylase (GAD)-65 along with anti-inflammatory cytokine IL-10 to newly diabetic NOD mice led to a significant diabetes reversal, which was fortified up to 67% when in combination with low-dose anti-CD3 antibody (195). Importantly, this effect that persisted after treatment discontinuation (195). *Clostridium butyricum* has been reported to protect against T1DM by modulating intestinal immune homeostasis and inducing pancreatic regulatory T cells (196). The development of autoimmune diabetes in NOD mice could be alleviated by taking a probiotic combination comprising five bacterial strains that acted by reducing gut permeability, inducing gut-homing Treg cells, and reducing Th1 polarization (197). *Akkermansia muciniphila*, a candidate of next-generation probiotics, appeared to slow the progression of T1DM in NOD mice (198, 199).

**Table 3:** Studies investigating the efficacy of probiotics in preventing and alleviating murine and human type 1 diabetes mellitus (T1DM).

**Subjects**	**Probiotic species/products**	**Probiotic dose**	**Probiotic supplementation time**	**Main observations**	**Reference(s)**
NOD mice	VSL#3	9 ×10^8^ CFU/d	3 times per week from 4 to 32 weeks of age	Reduced incidence of T1DM; reduced insulitis and a decreased rate of beta cell destruction; increased production of IL-10	(192)
NOD mice	VSL#3	14 mg/kg/d	3 times per week from 4 to 20 weeks of age	Protected from T1DM; altered microbiota composition; dampened intestinal inflammation; restored gut immune homeostasis; balanced the protective Teff/Treg cell in the gut mucosa	(193)
NOD mice	*Lactococcus lactis*	2 ×10^9^ CFU/d	5 times per week for 6 weeks	Reverted diabetes in NOD mice; increased frequencies of local Tregs	(194)
NOD mice	*Clostridium butyricum*	2.5 ×10^8^ CFU/kg/d	Once daily from 3 to 45 weeks of age	Prevented the onset of diabetes; induced pancreatic Treg cells	(196)
NOD mice	Immune regulation and tolerance 5 (IRT5)	10^9^ CFU/d	6 times a week for 36 weeks	Reduced incidence of T1DM; reduced gut permeability, increased gut-homing Treg cells; reduced Th1 polarization	(197)
Children	–	–	0–27 d of age	Decreased risk of islet autoimmunity	(200)
Children	*Lactobacillus rhamnosus*	10^9^ CFU/d	Once daily for 12 weeks	Increased circulating levels of tryptophan; decreased inflammatory cytokine production	(201)
Children	VISBIOME	1.1 ×10^11^ CFU/d	Once daily for 12 weeks	Reduced glycated hemoglobin; reduced total and bolus insulin requirements	(202)
Children	*Lactobacillus rhamnosus* and *Bifidobacter-ium* lactis	10^9^ CFU/d	Once daily for 24 weeks	No significant effect on pancreatic beta-cell function	(203)

*1. VSL#3 contained Bifidobacterium (Bifidobacterium longum, Bifidobacterium infantis, and Bifidobacterium breve), Lactobacillus (Lactobacillus acidophilus, Lactobacillus casei, Lactobacillus delbrueckii subsp. bulgaricus, and Lactobacillus plantarum), and Streptococcus salivarius subsp. thermophilus*.

*2. Immune Regulation and Tolerance 5 (IRT5) was a probiotic combination comprising Lactobacillus acidophilus, Lactobacillus casei, Lactobacillus reuteri, Bifidobacterium bifidium, and Streptococcus thermophilus*.

*3. VISBIOME contained Lactobacillus paracasei DSM 24733, Lactobacillus plantarum DSM 24730, Lactobacillus acidophilus DSM 24735, and Lactobacillus delbrueckii subsp. bulgaricus DSM 24734, Bifidobacterium longum DSM 24736, Bifidobacterium infantis DSM 24737, Bifidobacterium breve DSM 24732, and Streptococcus thermophilus DSM 24731*.

*4. –, not available*.

A prospective cohort study of 7,473 children showed that early probiotic supplementation could reduce the risk of islet autoimmunity in children at the highest genetic risk of T1DM (200). Another study of 61 young T1DM patients given either *Lactobacillus rhamnosus* GG or placebo daily for 12 weeks showed that intake of probiotics significantly increased circulating levels of tryptophan and decreased inflammatory cytokine production (201). The results of a recent randomized, double-blind, and placebo-controlled pilot study assessing treatment with a multispecies probiotic preparation, in 90 children with new-onset T1DM showed that better glycemic control and a decrease in insulin requirements (202). Notably, a double-blind, randomized controlled trial found that the intake of *Lactobacillus rhamnosus* GG and *Bifidobacterium lactis* Bb12 had no effect on beta-cell function in children newly diagnosed with T1DM (203).

Similar to AP, contradicting results have been obtained regarding the clinical efficacy of probiotics in management of T1DM. The discrepancies should be taken into account in future studies and in design of novel treatment strategies.

#### Pancreatic Cancer and Probiotics

Studies of the beneficial effects of probiotic consumption on pancreatic cancer seem to be just beginning, and most published works were conducted in animal models. Probiotic *Lactobacillus* could enhance gemcitabine-mediated antitumor efficacy in mice with pancreatic cancer (204). This finding is strengthened by another recently published study where authors further observed that probiotics could reduce gemcitabine-induced side effects by restoring a favorable microbiota (205). In addition, probiotic-treated super-charged NK cells could prevent the growth of pancreatic tumors through lysis and differentiation in humanized-BLT mice (206). The MAPK-p38 and transforming growth factor-β (TGF-β) signaling pathways have been hypothesized to be the mechanisms by which probiotics exert their anti-tumor effects (207, 208). Whether probiotics have a role in slowing or inhibiting the progression of pancreatic cancer needs to be further investigated and warrants future preclinical and clinical studies.

### Prebiotics

The ISAPP consensus statement (2016) defined the term prebiotics as “a substrate that is selectively utilized by host microorganisms conferring a health benefit,” including conjugated linoleic acids, polyunsaturated fatty acids, oligosaccharides such as fructooligosaccharides, inulin, galactooligosaccharides, mannanoligosaccharides, xylooligosaccharides, human milk oligosaccharides, phenolics, and phytochemicals (151). Administration of chitosan oligosaccharides to mice for 4 weeks prior to induction of SAP significantly reduced the severity of pancreatic injury by reducing oxidative stress and modulating the gut microbiota (209). Correlations between prebiotic fiber supplementation and hospital stay, duration nutrition therapy, acute phase response and overall complications were identified in patients with SAP in a randomized prospective double-blind controlled clinical trial (210). Inulin-type fructans were thought to play a role in preventing the development of AP and T1DM (211, 212). Supplementation of low-methoxyl pectin in NOD mice alleviated T1DM by increasing colonic SCFA production (213, 214). Similar results have been reported in a separate study investigating the effect of human milk oligosaccharides on T1DM in NOD mice (215). A randomized, placebo-controlled trial showed that the administration of prebiotics (oligofructose-enriched inulin) could improve glycemic control in children with T1DM (216). A series of investigations have also shown that a diet rich in resistant starch was significantly associated with decreased risk of experimental pancreatic malignancies (217, 218).

### Synbiotics

The definition of symbiotic is “a mixture comprising live microorganisms and substrate(s) selectively utilized by host microorganisms that confers a health benefit on the host,” according to the ISAPP consensus statement issued in 2019 (152). Enteral feeding with Synbiotic 2000 not only reduced organ dysfunctions in patients with SAP, but also improved intestinal barrier function (219, 220). Administration of synbiotics composed of *Lactobacillus casei, Lactobacillus rhamnosus, Lactobacillus acidophilus, Bifidobacterium bifidum*, and fructooligosaccharides in patients with CP increased the serum concentrations of hemoglobin, hematocrit, erythrocytes, total count of lymphocytes, magnesium, and albumin, and meanwhile reduced the total cholesterol values, without altering the nutritional status of the patients (221). In another single-blind prospective randomized control trial, synbiotics containing *Streptococcus faecalis, Clostridium butyricum, Bacillus mesentericus, Lactobacillus sporogenes*, and fructooligosaccharides significantly reduced septic complications, hospital stay, and antibiotic requirement in patients undergoing pancreatic surgery for CP (222). A randomized, double-blind, placebo-controlled trial showed that synbiotic supplementation in patients with T1DM might be effective in improving fasting blood glucose, hemoglobin A1c, insulin, hypersensitive C-reactive protein, and total antioxidant capacity (223).

### Postbiotics

The nomenclature of postbiotics was not clear until 2021 when the ISAPP defined a postbiotic as a “preparation of inanimate microorganisms and/or their components that confers a health benefit on the host” (153). Evidence is emerging that postbiotics have multiple health benefits and are considered to be the next horizon in microbial therapeutics and functional foods (224). In a cerulein-induced AP mouse, oral administration of probiotic *Lactobacillus brevis* SBL88-derived polyphosphate for 24 days prior to induction of AP mitigated the severity of AP, which was not only associated with modulation of the intestinal microbiome but also enhancement of the gut barrier integrity (225). A postbiotic, heat-killed *Lactococcus chungangensis* CAU 1447, could facilitate wound healing in type I diabetic mice; however, the authors did not investigate its effect on T1DM (226). Ferrichrome, derived from *Lactobacillus casei* ATCC334, has been shown to inhibit the growth of pancreatic cancer cells (227).

### Fecal Microbiota Transplantation

Fecal microbiota transplantation is a procedure in which stool from a healthy donor is placed into another patient's intestine (228). FMT to both antibiotic-treated mice and GF mice resulted in an aggravated AP (94). Heparanase-transgenic mice had more severe AP than wild-type mice; however, transfer of feces from the former to the latter worsened the disease (229). Parallels have also been observed in Western-type diet in combination with acute necrotizing pancreatitis (ANP) mice (230). However, a case report showed that FMT could be an effective therapeutic strategy in MSAP patients (231). The exact implication of FMT in the onset of pancreatitis remains to be proven in animal and human studies. In specific pathogen-free NOD mice, females have 1.3–4.4 times higher incidence of T1DM. Gavage transfer of gut microbiota from adult males to immature females altered the recipient's microbiota, elevated the testosterone, caused metabolomic changes, reduced islet inflammation and autoantibody production, and protected against T1DM (133). Transplanting fecal samples from diabetes-protected MyD88-deficient NOD mice to wild type female NOD/LtJ mice led to a delayed onset of diabetes and a reduced insulitis (119). Early-life antibiotic exposure not only perturbed the intestinal microbiota, but also accelerated the development of T1DM in the NOD mouse model; however, maternal cecal microbiota transfer to antibiotic-induced NOD mice restored the enhanced disease risk to baseline levels (232, 233). A randomized controlled trial published in 2021 found that FMT halted the onset of T1DM in human (234). Some other human FMT trials are ongoing for T1DM (NCT04124211; NCT04749030). A paucity of studies have investigated the application of FMT in pancreatic cancer, except those mentioned earlier.

In summary, research on the application of probiotics in pancreatic diseases has spawned a great deal of data; however, the data from prebiotics, synbiotics, postbiotics, and FMT is rather limited. Although heterogeneity exists in some studies, the application of probiotics, prebiotics, synbiotics, postbiotics, and FMT for improving pancreatic diseases remains promising.

## Future Strategies

Recent metagenomics and metabolomics studies have highlighted the complex interplay between the gut microbiome and pancreatic-related disorders. However, these approaches have several limitations. Firstly, they are association studies rather than causal research, which is needed to show whether alterations in the gut microbiome and its metabolites are a cause or a consequence of the disease. Secondly, although it is possible to describe the full spectrum of microorganisms through metagenomics studies, targeting strains like traditional culture approaches is difficult. Thirdly, metabolomics has almost become a golden standard used to depict metabolic profiles of the gut microbiome, but it is not easy to identify sources of specific metabolites on the species or strain level. To address these constraints, GF animal model and culturomics have become essential tools for exploring potential causality between host-microbial interactions (235, 236). The GF animal model is an attractive model, which is devoid of microorganisms *per se*. Excluding interference introduced by the host indigenous microbiome makes GF animal an indispensable key model for causality research. Culturomics has enabled laboratory cultivation and characterization of the vast majority of intestinal microbes, which is integral to research into microbiome (237). Additionally, application of other complementary technologies, such as metatranscriptomics and metaproteomics, will expand our understanding of the gut microbiome and its function.

Modulation of the gut microbiome by probiotics, prebiotics, synbiotics, postbiotics, and FMT has reinforced the potential for improving pancreatic diseases. However, animal and human data with respect to probiotics are equivocal in some cases, which could be due to several reasons. Firstly, discrepancies in probiotic strains, dosage, duration and frequency of administration in studies investigating effects of probiotic application are likely responsible for the inconsistent findings. Secondly, probiotics are supposed to act on the gut microbiome; however, the large variation in the indigenous gut microbial landscape between individuals is another major confounder. Consistency of the baseline microbiota should be taken into account given that environmental factors [such as diet (238–240), age (241), geography (241), and birth mode (242, 243)] that determine the gut microbiome differ between individuals. Finally, heterogeneity in the host *per se* exists. Collectively, these limitations should be taken into account in future study design (Box 1).

Box 1 **Strategies and considerations in design of microbiome and probiotics studies in the future.**

- Determining potential causality between host-microbial interactions by combining GF animal model, culturomics, and multi-omic technologies, e.g., metagenomics, metabolomics, metatranscriptomics, and metaproteomics.
- Assessing the consistency of the baseline microbiota prior to the start of preclinical and clinical trials.
- Constructing standardized protocols for trial conduction, e.g., probiotic strains, doses, duration and frequency of administration, to ensure comparability between studies and results generated by different laboratories.
- Constructing a standardized probiotic effect evaluation protocol to improve comparability of clinical outcomes between studies.

## Conclusions

There is growing appreciation of the fact that the gut microbiome imprints pancreatic diseases including pancreatitis, pancreatic cancer, and T1DM based on a multitude of preclinical and clinical studies. Evidence of the intestinal microbiome in driving pancreatic diseases further supports the significance of the extensive gut-pancreas crosstalk, leading to intense interest in microbiome characterization and engineering *via* application of probiotics, prebiotics, synbiotics, postbiotics, or FMT. Researches over the last decades have advanced our understanding of the relationship between the gut microbiome and pancreatic diseases. Such advent would also bring about novel strategies in improving pancreatic diseases *via* modulating the gut microbiome. Despite this breakthrough, potential causality between the gut microbiome and pancreatic diseases remains obscure. Efforts to untie the causality and microbiota-mediated mechanisms of pancreatic diseases have critical clinical implications, enabling us to better constitute a new and targeted approach to modulate the gut microbiome. Moreover, a body of preclinical research and high-quality clinical trials are needed to further elucidate mechanisms by which the gut microbiome influences pancreatic diseases, as well as mechanisms of probiotics, prebiotics, synbiotics, postbiotics, and FMT in improving disease outcomes. Ultimately, future development in the field will aim to harness microbiome engineering in clinical practice on a personalized level.

## Author Contributions

GG and HS: conceptualization. TZ: writing—original draft preparation. L-YK and ZS: writing—review and editing. All authors have read and agreed to the published version of the manuscript.

## Funding

This project was supported by the Science and Technology Major Projects of Inner Mongolia Autonomous Region (2021ZD0014) and China Agriculture Research System of MOF and MARA.

## Conflict of Interest

The authors declare that the research was conducted in the absence of any commercial or financial relationships that could be construed as a potential conflict of interest.

## Publisher's Note

All claims expressed in this article are solely those of the authors and do not necessarily represent those of their affiliated organizations, or those of the publisher, the editors and the reviewers. Any product that may be evaluated in this article, or claim that may be made by its manufacturer, is not guaranteed or endorsed by the publisher.
